# Effects of Tai Chi and Qigong on the mobility of stroke survivors: A systematic review and meta-analysis of randomized trials

**DOI:** 10.1371/journal.pone.0277541

**Published:** 2022-11-17

**Authors:** Moonkyoung Park, Rhayun Song, Kyoungok Ju, Jisu Seo, Xing Fan, Ahyun Ryu, YueLin Li, Taejeong Jang

**Affiliations:** 1 College of Nursing, Chungnam National University, Daejeon, Republic of Korea; 2 Department of Nursing, Woosuk University, Wanju, Republic of Korea; Prince Sattam Bin Abdulaziz University, College of Applied Medical Sciences, SAUDI ARABIA

## Abstract

**Background:**

Stroke survivors often experience impaired mobility and physical functions. Tai Chi and Qigong have been shown to have physical and psychological benefits for stroke patients.

**Purpose:**

To summarize the evidence on Tai Chi and Qigong for improving mobility in stroke survivors, specifically the ability to walk, dynamic balance, and activities of daily living (ADL).

**Methods:**

Independent searches of 16 electronic databases in English, Korean, and Chinese from their inception until December 2021 were conducted by two research teams. Methodological quality was assessed using Cochrane’s risk of bias tool 2.0. Comprehensive Meta-Analysis 3.0 software was used to calculate effect sizes with subgroup analysis and to assess heterogeneity and publication bias.

**Results:**

The meta-analysis included 27 randomized trials (18 with Tai Chi and 9 with Qigong) on stroke survivors (N = 1,919). None of the studies were considered at high risk of bias, about 70% had some concerns, and 30% were considered low risk. Meta-analysis of 27 randomized controlled trials with random-effects models indicated that Tai Chi and Qigong effectively improved mobility, specifically on the ability to walk (Hedges’g = 0.81), dynamic balance (Hedges’g = 1.04), and ADL (Hedges’g = 0.43). The effects of Tai Chi and Qigong were significant for short-term and long-term programs (Hedges’g 0.91 vs. 0.75), and when compared with active controls and no treatment group (Hedges’g 0.81 vs. 0.73).

**Conclusion:**

Tai Chi and Qigong performed for 12 weeks or less were effective in improving the mobility of stroke survivors. Further studies are warranted to assess whether Tai Chi and Qigong work best as an adjunct to rehabilitation, an effective alternative to rehabilitation or as a maintenance strategy, and whether the results could be further optimized by assessing different schools of Tai Chi and Qigong, different types of stroke patients, and different points in the post-stroke recovery process.

**PROSPERO registration number:**

This study has been registered on the UK National Institute for Health Research (http://www.crd.york.ac.uk/PROSPERO) PROSPERO registration number: CRD42020220277.

## Background

Stroke is a major worldwide cause of death and disability. Despite the declining stroke incidence, the aging population and accumulating risk factors contribute to an increased lifetime risk of stroke [1]. Stroke survivors often experience permanent disability and have difficulties performing activities of daily living (ADL) and walking [2]. Individuals with stroke also have reduced muscle strength and impaired mobility and physical functions [3], which reduces their quality of life [4].

Complex factors prevent stroke survivors from successfully surviving, and most risk factors are lifestyle-related and largely modifiable [5]. Physical activity, as one of the modifiable behavioral factors, has been shown as a rehabilitation approach and a significant component of comprehensive stroke rehabilitation programs to enhance balance, walking, and physical function among stroke survivors [6–8], while physical inactivity and physical deconditioning could contribute to worsening disability [9].

Tai Chi and Qigong (TCQ) are grounded in the principles of traditional Chinese medicine and have been described as equivalent in terms of essential forms and principles. Qigong is considered the ancient root of all traditional Chinese medicine practices [10], and many branches of Qigong have been developed for over 5,000 years. While hundreds of forms of Qigong have been developed in different regions of China, Tai Chi has become one of the best-known and most highly choreographed forms of performance in Qigong. A growing body of evidence supports the potential efficacy and safety of both Tai Chi and Qigong for various health conditions [11–13], specifically in promoting the motor function of stroke [14–16]. For these reasons, Tai Chi and Qigong are grouped together for this review as the equivalent intervention.

Recovery of mobility function is considered one of the main goals of stroke rehabilitation [17]. Although previous meta-analyses have strengthened the claim that TCQ is beneficial in stroke patients with related mobility function [18–22], only one of these studies performed a subgroup analysis showing that traditional Chinese exercises including TCQ were effective for limb function rehabilitation. At the same time, various types of TCQ with different intervention periods were applied [19]. Most of the other meta-analyses analyzed relatively small numbers of RCT studies, which prevented them from performing subgroup analyses to identify the effect on specific aspects of mobility, such as the ability to walk, dynamic balance, and activities of daily living (ADL).

Therefore, this study aimed to use a meta-analysis combined with recent clinical trials not included in previous reviews written in multi-languages. In addition, subgroup analyses were conducted based on outcome measures, duration of intervention, and control conditions to identify TCQ as an effective alternative intervention among stroke survivors.

## Methods

This review was registered in the PROSPERO database (PROSPERO Register code: CRD42020220277, http://www.crd.york.ac.uk/PROSPERO/), and reported based on the Preferred Reporting Items for Systematic Reviews and Meta-Analysis (PRISMA) guidelines [23].

### Eligibility criteria

This systematic review and meta-analysis included randomized controlled trials (RCTs) published in English, Korean, and Chinese. Participants were stroke patients with either cerebrovascular infarction or hemorrhage who had been discharged from hospitals and managed at rehabilitation or community health centers. The intervention group was those who applied Tai Chi or Qigong alongside conventional medication. There were no limitations on Tai Chi or Qigong intervention types, durations, or settings. The control group consisted of those who used other forms of physical activity, conventional or no treatment. Pretest and posttest data were also included.

The primary outcome of this review was mobility, defined as the ability to move freely and easily, which consisted of the objective measures of the ability to walk, dynamic balance, and ADL.

### Information sources and search strategies

A literature search was conducted between the year of inception and December 2021 on the following databases: Embase, PubMed, Cochrane Library, CINAHL, ScienceDirect, Ovid, DDOD for English, Research Information Sharing Service (RISS), Korean Studies Information Service System, National Digital Science Library, DBPIA, KoreaScholar, National Assembly Library of Korea, and Chinese National Knowledge Infrastructure (CNKI), Wanfang Data, and VIP for Chinese Science Journals Database.

To reduce publication bias, dissertations and conference proceedings were also searched by setting the search terms included in the title and abstract. In addition, the thesis and dissertation database were searched by DODD in English and Research Information Sharing Service (RISS) in the Korean database. In the Chinese database, the thesis and dissertation were automatically searched together during the general search. When duplicate studies were found, peer-reviewed articles were chosen over dissertations or conference proceedings.

We divided the search team into Korean, English, and Chinese teams in pairs. Each researcher independently searched their assigned databases to agree on a decision during the screening process and to minimize the number of missing studies. The search team used consistent search strategies. First, a search strategy was established using English MeSH and Emtree search terms. The Chinese database search strategy was then based on confirming it with the Chinese team and translating it into Chinese terms. In the Korean database, MeSH terms and corresponding keywords in Korean were used. All search strategies were established by sharing among the research teams, and progress was checked and shared through research meetings. Additional manual searches were conducted on Google Scholar and the references in the retrieved articles. MeSH and Emtree search terms were used, with Boolean operators used to combine these terms. S1 Table lists the search strategies for the English databases.

CNKI terms were the main terms for searching Chinese databases, including “Taichi (太极)” OR “Qigong (气功)” OR “Taiji Quan (太极拳)” OR “Baduan jin (八段锦)” (S2 Table). All articles selected from each database were screened for duplicates and managed using EndNote X9.3. Microsoft Excel was used to summarize the following characteristics of the included studies: year of publication, language, population, intervention type, outcome variables, and comparison groups.

### Risk of bias assessment

Two teams of reviewers independently assessed the bias risk of each selected English and Chinese study according to Cochrane’s risk of bias tool 2.0 (Cochrane RoB 2.0) [24]. Several issues arose when applying the original Cochrane RoB version, including interrater inconsistency in some domains, potential misunderstandings of selective reporting domains, and other types of bias. The updated Cochrane RoB 2.0 contained five domains: bias arising from the randomization process, bias in deviations from intended interventions, bias from missing outcome data, bias in outcome measurements, and bias in reported-result selection. The judgment on bias risk arising from each domain was made by an algorithm, leading to final decisions on ‘low-risk bias’, ‘some concerns’, or ‘high-risk bias’ [24]. Any discrepancy between two independent reviewers [JS&AR for English, KJ&TJ for Korean, FX&YL for Chinese] was discussed among the reviewers or a third expert reviewer [RS for the Korean or English team, MP for the Chinese team] if necessary to generate consensus.

### Data extraction and analysis

Comprehensive Meta-Analysis (version 3, Biostat, USA) was used to combine the effect sizes and assess heterogeneity and publication bias. The analyzed data included the sample description (sample size, and Tai Chi or Qigong intervention), duration (duration of one session, frequency, and intervention length), mobility outcome measure (the ability to walk, dynamic balance, and ADL), and control condition (active or no-treatment). As a mobility outcome, the ability to walk was measured using Timed Up and Go (TUG), gait (a subscale of the Short Physical Performance Battery [SPPB]), Dynamic Gait Index, functional ambulation category, computerized gait analysis, 10-m walk tests, 6-min walk tests, and 2-min step tests. Dynamic balance was measured using the Berg Balance Scale (BBS), and ADL was measured using the Barthel Index (BI) or modified Barthel Index (MBI) in all included studies.

Two independent researchers extracted and confirmed all available statistical data for the included studies and then entered them into an Excel spreadsheet. Quantitative data extraction included pre- and post-test data means and standard deviations, the mean and standard deviation of each group’s changed scores, and the sample size of each group. Hedges’ g was used to quantify effect sizes and 95% confidence intervals (CIs). A random-effects model was used for the analysis so that the results of this analysis could be compared with similar studies [25]. A subgroup analysis was conducted on intervention duration (less than 12 weeks as short-term vs. 12 weeks or longer as long-term), control condition (active control vs. no-treatment control), and outcome measures (the ability to walk vs. dynamic balance vs. ADL). The effect-size direction was determined by the difference between mean values for the experimental and control groups.

Heterogeneity was tested using 95% prediction intervals (PIs) computed using the CMA prediction intervals program [26]. Multiple control groups or outcomes included in single studies were entered separately when reporting mean effect sizes [27]. Publication bias was assessed using funnel plots [27] and Egger’s regression test (p >.05) [28].

## Results

### Study selection

**Fig 1:**
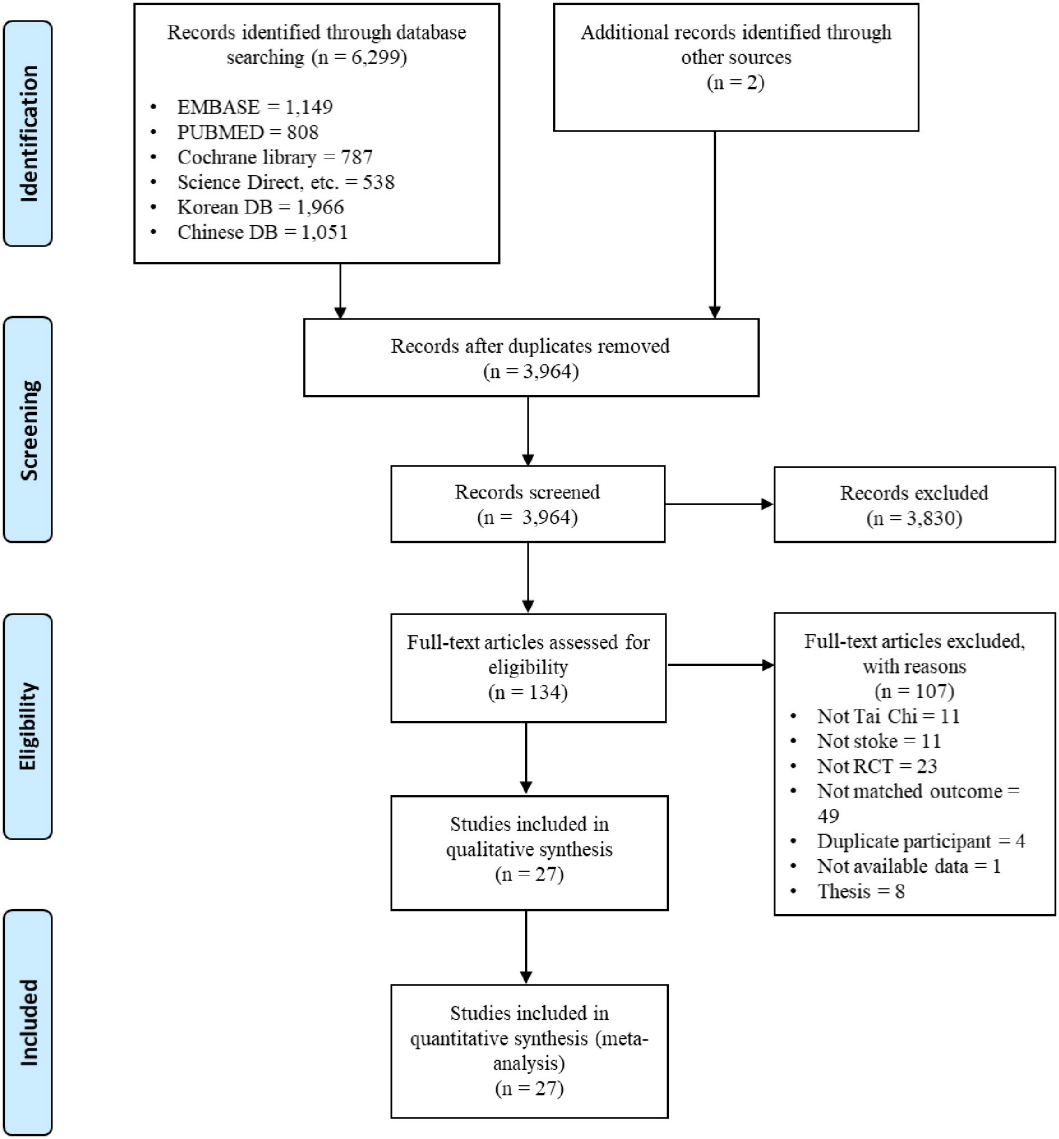
Flow diagram of the study selection process.

As shown in Fig 1, selecting literature for the qualitative and quantitative analyses was performed in line with the Cochrane guidelines and reported using PRISMA [23]. Of the 6,299 citations identified in the databases until December 2021, 3,282 were from Embase and other international web-based databases, 1,966 were from Korean databases, and 1,051 were from Chinese databases. Manual searches identified two additional citations. Duplicate citations were removed by reviewing titles and abstracts based on the inclusion criteria. After initial screening, the full texts of 134 articles were reviewed, and those without RCTs (k = 23), TCQ (k = 11), not matched mobility outcomes (k = 49), those with duplicate participants (k = 4), no data (k = 1) and not peer-reviewed studies (k = 8) were excluded. Qualitative synthesis and quantitative analysis were conducted on 27 studies (Fig 1).

### Quality of trials and bias risk

Bias risk was assessed using Cochrane RoB 2.0 to determine the bias level of each domain via an algorithm [24]. The domain-level judgments of bias risk were determined as a low bias risk if all domains had a low risk, some concerns if at least one domain had concerns, or a high bias risk if at least one domain had a high risk. A randomization process was described in all studies, but only seven were considered to have a low bias risk (29.6%). In comparison, there were some concerns about most studies (70.4%) due to a lack of a specific description of concealed or random allocation sequencing. No study suggested a baseline imbalance, which was the criterion for a high bias risk in the randomization process. For bias due to deviations from the intended intervention, 26 studies had a low bias risk (96.3%), and there were some concerns about one study (3.7%). Most studies involving Tai Chi or Qigong did not apply blinding to participants and staff; however, no deviation from the intended intervention was suspected in the experimental context.

**Fig 2:**
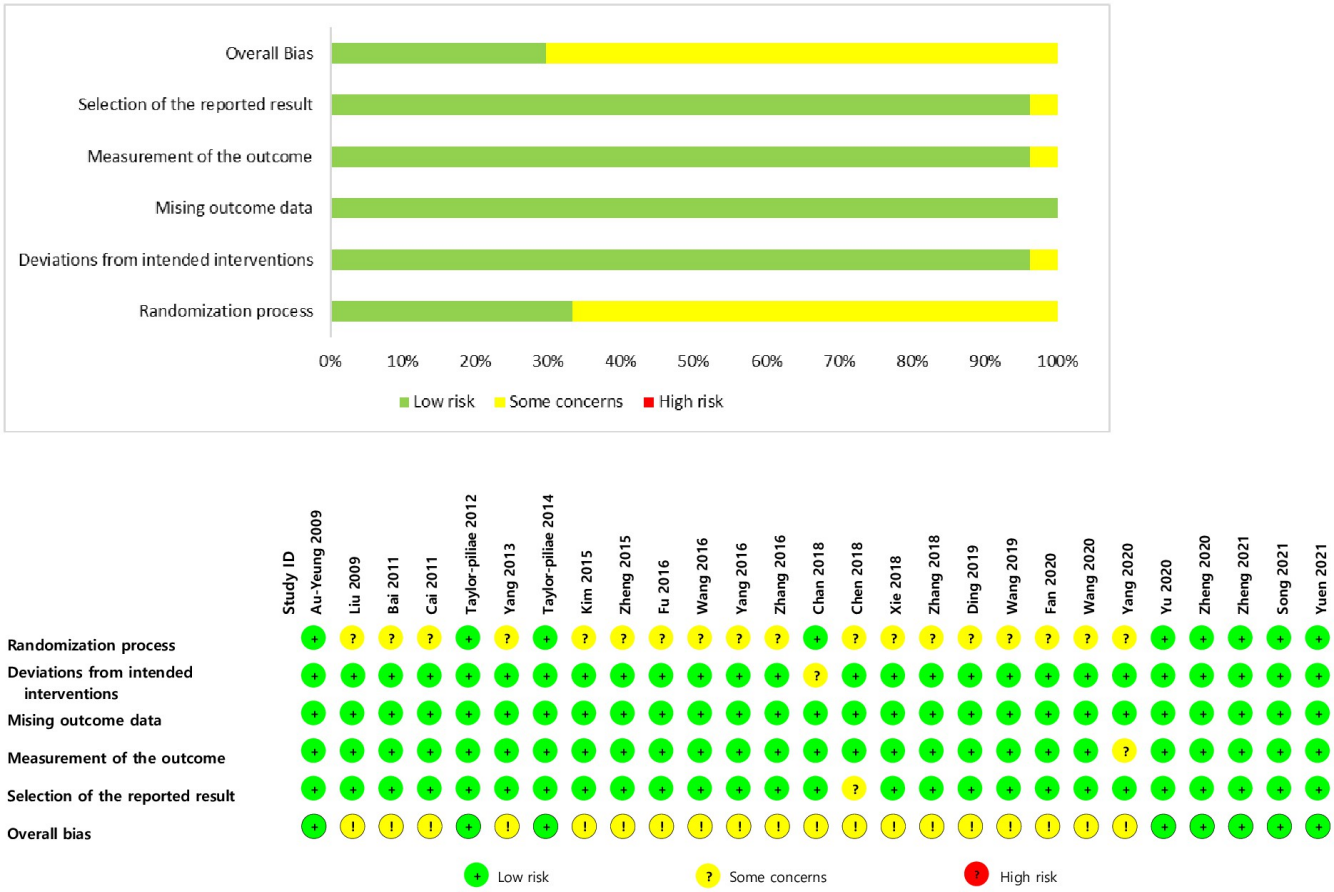
Assessment of the bias risk in the included studies.

In the domains of bias risk due to missing outcome data and outcome measurements, there were some concerns about only one study (3.7%), with most studies considered a low bias risk. Some of the study outcome measurements were conducted with blinding (k = 13), not specified (k = 8), or no blinding (k = 6), but mostly objective or valid measures were used as study outcomes. Regarding the bias risk in the selection of the reported results, there were some concerns about three studies (3.7%) and most studies (96.3%) had a low bias risk. The overall assessment indicated that there were some concerns about 70.4% of the studies, 29.6% had a low bias risk, and no high bias risk (Fig 2).

#### Modality and medium

The main characteristics of the 27 RCTs (18 using Tai Chi [14, 16, 29–44] and 9 using Qigong [45–53] are listed in Table 1. These studies were conducted in China (k = 23), USA (k = 2), and Korea (k = 2). The intervention settings for stroke patients were the hospital outpatient (k = 19) and community (k = 8). The sample size of these studies ranged from 16 to 244 (1,919 total stroke patients) with a mean age range of 45 to 69 years. About 62.1% of the participants were male.

**Table 1:** Characteristics of the included studies.

Study, year	Country	Setting	Language	Participants				Intervention			Comparison	Outcome measure	Safety monitoring
				E (M:F)	C (M:F)	Disease-related characteristics	Mean age (years) ^†^	Type	Intensity (per week)	Duration (weeks)			
Au-Yeung, 2009	Hong Kong	Community	English	59 (33:26)	55 (33:22)	Stroke time >6months	63.4±10.7	Tai Chi, Sun style	1h×1 (+3h self-practice)	12	Stretching and education	TUG	Harness during test
Liu, 2009	China	Community	Chinese	24 (14:10)	24 (11:13)	Unilateral hemiplegia	52.1±14.1	Tai Chi, Yang style	30min×1 (+6×self-training)	12	Home rehabilitation training	BBS	Caregiver support
Bai, 2011	China	Hospital	Chinese	30 (22:8)	30 (20:10)	Ischemic: 61.7% Hemorrhage: 38.3%	53.7±4.5	Qigong (Baduanjin)	20min×14 (2 sessions/day)	6	Balance training	BBS	NR
Cai, 2011	China	Community	Chinese	30 (20:10)	30 (23:7)	Stroke time ≥6 months	60.3±10.5	Sitting Qigong (Baduanjin)	30min×4/5	12	No treatment	BI	NR
Taylor-Piliae, 2012	USA	Hospital (OPD)	English	16 (10:6)	12 (7:5)	Stroke time ≥3months	69.3±11.0	Tai Chi, Yang style	1h×3	12	No treatment	SPPB (gait), 2-min step test	No AEs
Yang, 2013	China	Hospital	Chinese	50 (35:15)	50 (31:19)	First stroke	54.3±13.8	Tai Chi, not specified	45min×7	4	Balance training	BI, BBS	Protect from falls
Taylor-Piliae, 2014	USA	Community	English	53 (34:19)	AC: 44 (20:24) UC: 48 (23:25)	Ischemic: 66% Hemiparesis: 73%	69.9±10.0	Tai Chi, Yang style	1h×3	12	SilverSneakers or no treatment	SPPB (gait), 2-min step test	Monitored
Kim, 2015	Korea	Hospital	English	11 (7:4)	11 (6:5)	Stroke	53.5±11.5 (E) 55.2±10.2 (C)	Tai Chi, Yang style	1h×2	6	Physical therapy	Dynamic gait index, 10MWT, TUG	NR
Zheng, 2015	China	Hospital	Chinese	51 (27:24)	55 (31:24)	Ischemic stroke	59.0±13.0	Tai Chi, not specified	30min×14 (2 sessions/day)	48	Routine rehabilitation training	ADL (NI)	NR
Fu, 2016	China	Hospital	Chinese	30 (19:11)	30 (18:12)	Stroke time ≤3months	59.7±7.6	Tai Chi, Yang style	40min×6	8	No treatment	BBS, 10MWS	Assist patients
Wang, 2016	China	Community	Chinese	14 (9:5)	16 (14:2)	Stroke	45–75	Tai chi, Yun Shou style	20min×5	12	Balance training	BBS, gait analysis	NR
Yang, 2016	China	Hospital	Chinese	26 (17:9)	21 (14:7)	Stroke time ≤3months	51.4±15.6	Tai Chi, not specified	40min×3	8	No treatment	BBS	NR
Zhang, 2016	China	Hospital	Chinese	31 (17:14)	31 (18:13)	Stroke time 1–3months	55.1±4.8	Qigong (Baduanjin)	20min×10 (2 sessions/day)	12	Balance training	BBS	NR
Chan, 2018	Hong Kong	Community	English	15 (9:6)	AC: 17 (10:7) UC: 15 (8:7)	Stroke	63.0 ± 7.0	Tai Chi, Yang style	1h×2	12	Conventional exercise or no treatment	Gait analysis	No AEs
Chen, 2018	China	Hospital	English	8	8	Stroke time ≥3 months	50–75	Tai Chi, not specified	NI	24	No treatment	BBS	NR
Xie, 2018	China	Hospital	English	120 (83:37)	124 (99:35)	Ischemic: 74.2% Hemorrhage: 25.8%	60.9±8.7	Tai Chi, Yang style	1h×5	12	Balance training	BBS, TUG, MBI	No AEs
Zhang, 2018	China	Hospital	English	45 (27:18)	45 (25:20)	Ischemic: 60.0% Hemorrhage: 40%	63.7±6.8	Tai Chi, Yang style	40min×5	48	No treatment	BI	NR
Ding, 2019	China	Hospital (OPD)	Chinese	57 (33:24)	56 (31:25)	Ischemic: 46.9% Hemorrhage: 53.1%	55.4±4.7 (E) 56.3±3.2 (C)	Qigong (Baduanjin)	20min×5	4	Balance training	BBS	Monitored
Xie, 2019	China	Hospital	Chinese	30 (13:7)	20 (12:8)	Ischemic: 50% Hemorrhage: 50%	51.1±12.9 (E) 53.9±13.0 (C)	Qigong (Baduanjin)	50min×5	3	No treatment	BI, BBS, 6MWT	Monitored
Fan, 2020	China	Hospital	Chinese	43 (29:14)	43 (30:13)	Ischemic: 61.6% Hemorrhage: 38.4%	63.4±5.0 (E) 63.8±5.3 (C)	Tai Chi, not specified	1.5h×3	12	No treatment	BBS, TUG, 6MWT	Monitored
Wang, 2020	China	Hospital	Chinese	30 (16:14)	30 (17:13)	Stoke	55.1±6.3 (E) 55.9±6.2 (C)	Qigong (Baduanjin)	12min×5	4	No treatment	MBI	NR
Yang, 2020	China	Hospital	Chinese	30 (18:12)	30 (14:16)	Ischemic: 73.3% Hemorrhage: 26.7%	64.0±3.9 (E) 62.9±4.7 (C)	Tai Chi, Yang style	30min×2	6	No treatment	BI	Assist patients
Yu, 2020	China	Community	English	35 (21:14)	36 (20:16)	Stroke time ≥3months Ischemic: 57.7% Hemorrhage: 42.3%	63.0±8.9 (E) 58.7±9.7 (C)	7 step forms Tai Chi from 24-form Tai Chi	40min×3	12	Conventional rehabilitation program	Gait analysis, BBS	No AEs
Zheng, 2020	China	Community	English	24 (19:5)	24 (22:2)	Stroke time >3months (first ever stoke) Ischemic: 47.9% Hemorrhage: 52.1%	61.6±9.2 (E) 62.8±6.4 (C)	Qigong (Baduanjin)	40min×3	24	Routine rehabilitation treatment	MBI	No AEs
Zheng, 2021	China	Hospital	English	30 (24:6)	30 (19:11)	Stroke time <2months (first ever stoke) Ischemic: 78.3% Hemorrhage: 21.7%	63.5±10.4 (E) 67.2±9.2 (C)	Qigong (Liuzijue)	45min×1	3	Conventional respiration training	BBS, MBI	NR
Song, 2021	Korea	Hospital (OPD)	English	18 (10:8)	16 (11:5)	Ischemic: 58.8% Hemorrhage: 41.2%	58.7±17.1 (E) 57.1±10.7 (C)	Modified Tai Chi	50min×2	24	Symptom management program	BBS, FAC, K-MBI	No AEs
Yuen, 2021	Hong Kong	Hospital (OPD)	English	29 (15:14)	29 (14:15)	Stroke time >3months (first ever stoke) Ischemic: 62.1% Hemorrhage:37.9%	63.1±10.6 (E) 62.0±13.1 (C)	Qigong (Baduanjin)	50min×3	16	Stretching training	TUG, MBI	No AEs

^**†**^Data are mean±standard deviation or range values.

Abbreviations: M, males; F, females; MBI, modified Barthel Index; BBS, Berg Balance Scale; TUG, Timed Up and Go; BI, Barthel Index; SPPB, Short Physical Performance Battery; E, experimental group; C, control group; 10MWT, 10-m walking test; ADL, activities of daily living; AC, active control group; UC, no-treatment group; OPD, outpatient department; 10MWS, 10-m maximum walking speed; 6MWT, 6-min walking test; FAC, Functional Ambulation Category; K-MBI, Korean Version of the Modified Barthel Index; AEs, adverse events; NR, not reported

Stroke diagnoses consisted of ischemic or hemorrhagic infarction, including either hemiparesis or hemiplegia. Most studies did not include stroke duration as an inclusion criterion, with patients recruited within 3 months after the diagnosis in four studies, longer than 3 months in five studies, and longer than 6 months in two studies.

The intervention types varied for TCQ, while Yang style Tai Chi (k = 9) and Baduanjin Qigong (k = 8) were mostly applied. The intervention was defined as short term (k = 11) when performed for less than 12 weeks, with ranges of 2–14 sessions and 1–5 hours per week. The other 17 studies applied long-term interventions (12 weeks or longer), with ranges of 12–48 weeks, 2–14 sessions per week, and 20–90 min per session. The comparison groups were either a no-treatment control (received usual or no treatment) or an active control (received rehabilitation training, acupuncture, a national exercise program [e.g., the SilverSneakers app], or balance training).

#### Safety monitoring

No adverse events (AEs) occurred during the TCQ intervention in 7 of the 27 included studies [14, 16, 29, 31, 42, 50, 51], while nine studies implemented safety monitoring by therapists, staff, or caregivers [30, 32, 34, 36, 38, 39, 41, 45, 49]. Neither safety monitoring nor AEs during the program was reported for 59.3% (k = 16) of the studies.

### Meta-analysis: Synthesis of results

#### Primary outcome: Total effect on mobility

**Fig 3:**
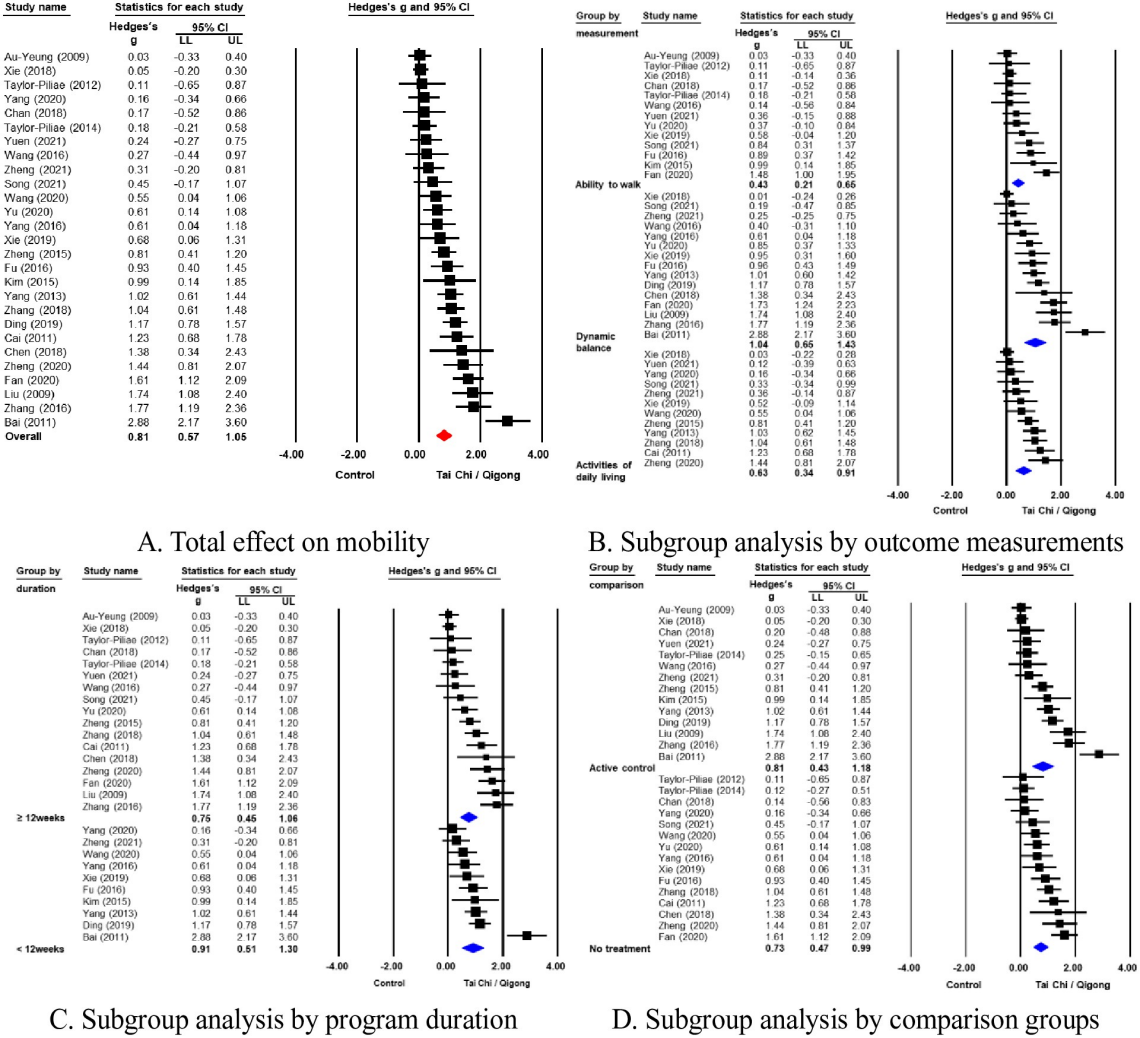
Forest plots of the effects of Tai Chi and Qigong on mobility.

The meta-analysis of 27 RCTs with random-effects models indicated that TCQ was effective in improving the mobility of stroke survivors (Hedges’ g = 0.81, 95% CI = 0.57 to 1.05). Potential heterogeneity across studies was indicated by a 95% prediction interval (PI) of –0.39 to 2.00 (Fig 3A). Publication bias was suspected based on asymmetric funnel plots and Egger’s regression test (p = .018) (S1 Fig).

### Subgroup analysis

1. Subgroup analysis by the ability to walk, dynamic balance, and activities of daily living
  Mobility was assessed as an outcome measure using the ability to walk (k = 15), dynamic balance (k = 15), or ADL (k = 12). Hedges’ g was calculated for each mobility measure: the ability to walk, dynamic balance, and ADL (Fig 3B).
  The ability to walk was assessed in 15 studies using TUG, gait analysis, 2-min step tests, 10-m walking tests, and 6-min walking tests. The significant effect of TCQ on the ability to walk was indicated by a Hedges’ g of 0.43 (95% CI = 0.21 to 0.65; 95% PI = –0.35 to 1.20). No publication bias was suspected based on funnel plots and Egger’s regression test (p = .176) (S1 Fig).
  Dynamic balance was measured using BBS in all included studies, and a significant large effect size of TCQ on balance (k = 15) was indicated (Hedges’ g = 1.04; 95% CI = 0.65 to 1.43; 95% PI = –0.54 to 2.62), with potential heterogeneity (Fig 3B). Publication bias was suspected based on funnel plots and Egger’s regression test (p = .024) (S1 Fig).
  ADL was measured using BI or MBI. The significant effect size of TCQ on ADL (k = 12) was indicated by a Hedges’ g of 0.63 (95% CI = 0.34 to 0.91; 95% PI = –0.38 to 1.63). The symmetrical funnel plot and Egger’s regression test (p = .110) suggested that publication bias was not present (S1 Fig).
2. Subgroup analysis by program duration
  A subgroup analysis was conducted based on program duration, presented as either short term (k = 10) or long term (k = 17). The effect sizes of TCQ on mobility were indicated by Hedges’ g values of 0.91 (95% CI = 0.51 to 1.30; 95% PI = –0.47 to 2.28) and 0.75 (95% CI = 0.45 to 1.06; 95% PI = –0.47 to 2.28) for short- and long-term interventions, respectively. The effect of TCQ on mobility was significant regardless of the duration of intervention, but there is no significant difference in the effect sizes between short- and long-term interventions (Q = 0.37, p = .544) (Fig 3C). The prediction interval indicated the presence of heterogeneity among the included studies. No publication bias was considered based on funnel plots and Egger’s regression test for the short-term (p = .538), but publication bias was suspected in the long-term with significant Egger’s regression test (p = .038) (S1 Fig).
3. Subgroup analysis according to comparison groups
  A subgroup analysis was conducted between active control (k = 14) and no-treatment control (k = 15). The effect sizes of TCQ on mobility were indicated by Hedges’ g values of 0.81 (95% CI = 0.43 to 1.18; 95% PI = -0.69 to 2.31) and 0.73 (95% CI = 0.47 to 0.99; 95% PI = –0.23 to 1.69) for active control and no treatment control, respectively. The effect sizes of TCQ on mobility were significant regardless of the types of comparison groups, but no significant difference was found between active control and no-treatment control groups (Q = 0.12, p = .730) (Fig 3D). The funnel plot was symmetrical, but significant Egger’s regression tests (p = .038) suggested publication bias for active control. There was no publication bias for no-treatment control (p = .687) (S1 Fig).

## Discussion

Meta-analysis of 27 RCTs with 1,919 subjects found TCQ improved mobility, including the ability to walk, dynamic balance, and ADL in stroke patients. The effect size of the random effect model remained significant for different intervention duration, and even when compared with active control groups such as physiotherapy, balance training, or combined exercise programs.

The ability to walk was measured in our included studies using TUG, PPB, gait analysis, and walking tests, with PIs of –0.66 to 1.32. While some concerns about heterogeneity exist, these findings indicate the potential benefits of TCQ in improving the ability to walk (Hedges’ g = 0.43) among stroke survivors. A previous meta-analysis involving five RCTs assessed the ability to walk using TUG and SPPB and similarly found a small effect size [20].

Our analysis of dynamic balance based on 20 studies indicated the inclusion of TCQ in stroke rehabilitation programs would improve dynamic balance (Hedges’ g = 1.04) among stroke survivors for a duration less than 12 weeks. Previous meta-analyses have supported that TCQ affects dynamic balance when performed two or three times weekly for 6–12 weeks [20, 54]. The effect of TCQ on ADL (Hedges’ g = 0.63) in our subgroup analysis was also supported by a previous meta-analysis of 31 RCTs with traditional Chinese exercise as an intervention (MD = 15.60 on the BI scale) [19].

However, the effects of TCQ on mobility have varied between outcome measures or intervention dose [54]. Our meta-analysis indicated that TCQ effectively improved the ability to walk with a relatively small effect size (Hedges’ g = 0.43), which became insignificant in the subgroup analysis after adjusting for intervention durations. According to a meta-analysis of 19 RCTs with stroke patients, Tai Chi was effective in improving mobility when performed in sessions lasting 30–60 min for five or more times each week [54]. A review of 75 RCTs on stroke survivors also suggested that exercise interventions were effective in improving mobility (1) regardless of whether the duration was less than 12 weeks or at least 12 weeks, (2) with intensity at 60–80% of maximum heart rate, and (3) when performed regularly (mostly 3–5 days/week) and progressively [55]. This information could be useful when determining the optimal intervention dose for improving mobility in stroke survivors. An optimal dose of the training content would be more beneficial for stroke survivors than increasing the intensity or duration [55].

Multiple clinical studies have considered TCQ safe and feasible interventions for improving mobility in stroke survivors [16, 29, 31, 32]. Adverse events (AEs) reporting within clinical trials is essential in assessing intervention safety, preferably via full descriptions of AEs monitoring protocols and/or specific AEs reported by the participants. Less than half the studies in this meta-analysis mentioned safety monitoring procedures (K = 12), and only 5 reported no AEs. This is slightly better than the 35% reporting rate identified in a previous study [56].

Some strengths and limitations should be considered when interpreting the results. The strengths of this study would be that it drew from three different language databases, including from countries where TCQ is widely practiced and that the quality of the included trials is fairly good—with none at high risk of bias and almost 30% at low risk of bias. In addition, with the exception of walking ability, there appeared to be a low possibility of publication bias when assessed for the different components of mobility.

The main limitation of this study was the heterogeneity among the studies due to the wide range of stroke-related symptoms and rehabilitation stages. The types of intervention, although all based on traditional Chinese principles, were different in timing, intensity, and duration. Moreover, mobility was assessed using various outcome measurement methods. A subgroup analysis was performed, which defined mobility using the ability to walk, dynamic balance, and ADL, yet the PI was still large (from –0.54 to 2.62), indicating heterogeneity across the included studies.

### Implication

Further studies are warranted to assess whether TCQ works best as an adjunct to rehabilitation, an effective alternative to rehabilitation or as a maintenance strategy, and whether the results could be further optimized by assessing different schools of TCQ, different types of stroke patients, and different points in the post-stroke recovery process. Although TCQ has been considered safe and feasible for stroke survivors, all future trials should have careful safety monitoring plans in place and report adverse events on the findings.

## Conclusion

Our review suggests that TCQ was effective in improving mobility including the ability to walk, dynamic balance, and ADL among stroke survivors for programs of both shorter and longer duration. This effect of TCQ remained significant when compared with other alternative interventions. As an effective alternative to rehabilitation, TCQ could effectively applied to stroke survivors to promote functional recovery through improving the ability to walk, dynamic balance, and ADL. The heterogeneity of the included studies should be considered.

## Supporting information

S1 Checklist PRISMA 2009 checklist. (DOCX)

S1 Fig Funnel plots of the effects of Tai Chi and Qigong on mobility. (TIF)

S1 Table Examples of search strategies in PubMed and Embase. (DOCX)

S2 Table Korean and Chinese search terms. (DOCX)
