# Supplementary figures and images for: Effects of Tai Chi and Qigong on the mobility of stroke survivors: A systematic review and meta-analysis of randomized trials
Source: PLoS One. 2022 Nov 17;17(11):e0277541. doi: 10.1371/journal.pone.0277541 (PMC9671349; doi:10.1371/journal.pone.0277541)

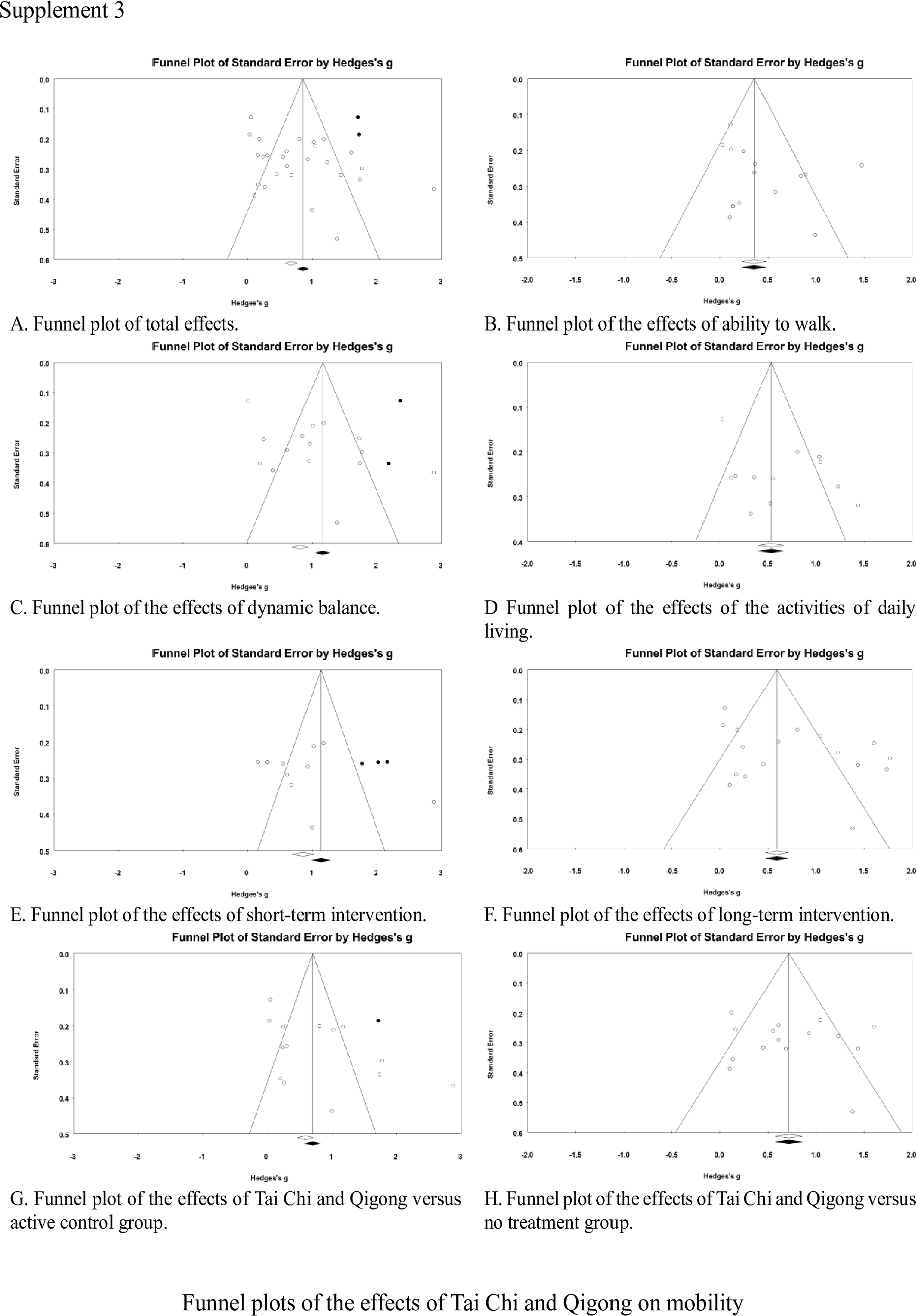

Supplement: S1 Fig — (TIF) [file pone.0277541.s002.tif]
