# Supplementary material for: Effects of Tai Chi and Qigong on the mobility of stroke survivors: A systematic review and meta-analysis of randomized trials
Source: PLoS One. 2022 Nov 17;17(11):e0277541. doi: 10.1371/journal.pone.0277541 (PMC9671349; doi:10.1371/journal.pone.0277541)
Supplement: S1 Table — (DOCX) [file pone.0277541.s003.docx]

Supplement 1. Examples of search strategies in PubMed and Embase

| Database | Search number | Query | Results |
| --- | --- | --- | --- |
| PubMed | #1 | ((((((((((((Tai ji[MeSH Terms]) OR (Qigong[MeSH Terms])) OR (Tai chi[Title/Abstract])) OR (taiji[Title/Abstract])) OR (taichi[Title/Abstract])) OR (Tai ji[Title/Abstract])) OR (T'ai chi[Title/Abstract])) OR (Tai-ji[Title/Abstract])) OR (Taijiquan[Title/Abstract])) OR (taijichuan[Title/Abstract])) OR (Qigong[Title/Abstract])) OR (Chi Kung[Title/Abstract])) OR (Ch'i Kung[Title/Abstract]) | 3,035 |
|  | #2 | ((((((random allocation[MeSH Terms]) OR (controlled clinical trials as topic[MeSH Terms])) OR (controlled clinical trial[MeSH Terms])) OR (random*[Title/Abstract])) OR (RCT[Title/Abstract])) OR (controlled clinical[Title/Abstract])) OR (clinical trial*[Title/Abstract]) | 1,725,082 |
|  | #3 | #1 AND #2 | 1395 |
|  | #4 | (((((((((((((((((((movement[MeSH Terms]) OR (physical fitness[MeSH Terms])) OR (activities of daily living[MeSH Terms])) OR (Mobility limitation[MeSH Terms])) OR (Postural balance[MeSH Terms])) OR (functional status[Title/Abstract])) OR (physical performance[Title/Abstract])) OR (physical perform*[Title/Abstract])) OR (physical function*[Title/Abstract])) OR (walk*[Title/Abstract])) OR (mobility[Title/Abstract])) OR (ambulat*[Title/Abstract])) OR (activit*[Title/Abstract])) OR (motor[Title/Abstract])) OR (timed up[Title/Abstract] AND go[Title/Abstract])) OR (locomotor[Title/Abstract])) OR (postur*[Title/Abstract] AND balance[Title/Abstract])) OR (postur*[Title/Abstract] AND control*[Title/Abstract])) OR (equilibrium*[Title/Abstract])) OR (dynamic balance[Title/Abstract]) | 4,547,093 |
|  | #5 | #3 AND #4 | 808 |
| Embase | #1 | 'tai chi'/exp OR 'qigong'/exp OR taiji:ti,ab,kw OR taichi:ti,ab,kw OR 'tai chi':ti,ab,kw OR 'tai ji':ti,ab,kw OR taijiquan:ti,ab,kw OR taijichuan:ti,ab,kw OR qigong:ti,ab,kw OR 'chi kung':ti,ab,kw | 5,406 |
|  | #2 | 'controlled clinical trial'/exp OR random*:ti,ab,kw OR rct:ti,ab,kw OR 'controlled clinical':ti,ab,kw OR 'clinical trial*':ti,ab,kw | 2,416,967 |
|  | #3 | #1 AND #2 | 2,113 |
|  | #4 | 'walking'/exp OR 'body equilibrium'/exp OR 'physical mobility'/exp OR 'fitness'/exp OR 'functional status'/exp OR 'functional status assessment'/exp OR 'physical performance'/exp OR 'physical perform*':ti,ab,kw OR 'physical function*':ti,ab,kw OR walk*:ti,ab,kw OR mobility:ti,ab,kw OR ambulat*:ti,ab,kw OR activit*:ti,ab,kw OR motor:ti,ab,kw OR ('timed up':ti,ab,kw AND go:ti,ab,kw) OR locomotor:ti,ab,kw OR (postur*:ti,ab,kw AND balance:ti,ab,kw) OR (postur*:ti,ab,kw AND control*:ti,ab,kw) OR equilibrium*:ti,ab,kw | 5,413,563 |
|  | #5 | #3 AND #4 | 1,149 |
