# Supplementary material for: Effects of Tai Chi and Qigong on the mobility of stroke survivors: A systematic review and meta-analysis of randomized trials
Source: PLoS One. 2022 Nov 17;17(11):e0277541. doi: 10.1371/journal.pone.0277541 (PMC9671349; doi:10.1371/journal.pone.0277541)
Supplement: S2 Table — (DOCX) [file pone.0277541.s004.docx]

Supplement 2. Korean and Chinese search terms.

| Language | Query |
| --- | --- |
| Korean | (“Tai Chi (태극권 or 타이치)” OR “Qigong (기공)”) AND “randomized(무작위 or random*)” |
| Chinese | “Tai Chi (太极)” OR “Qigong (气功)” OR “Taiji Quan (太极拳)” OR “Baduanjin (八段锦).” |
